# Radiological and FDG-PET imaging features of Epstein–Barr virus–positive primary central nervous system lymphomas

**DOI:** 10.1007/s00415-026-13974-z

**Published:** 2026-07-06

**Authors:** Nina Schulz, Dario Herrán de la Gala, Laura Rozenblum, Patrizia Lazzari, Véronique Morel, Ines Boussen, Julie Abraham, Marie Le Cann, Carole Soussain, Marie Dorel, Renata Ursu, Delphine Leclercq, Marie Blonksi, Karima Mokhtari, Bertrand Mathon, Khe Hoang-Xuan, Sylvain Choquet, Caroline Houillier, Lucia Nichelli

**Affiliations:** 1 Neurology Department, Bicêtre Hospital, AP-HP, Le Kremlin-Bicêtre, France; 2 Neuroradiology Department, Pitié-Salpêtrière University Hospital, AP-HP, Paris, France; 3 Department of Nuclear Medecine, Pitié-Salpêtrière University Hospital, AP-HP, Paris, France; 4 Hematology Department, Pitié-Salpêtrière University Hospital, Pierre et Marie Curie University, GRC11.GRECHY, AP-HP, Paris, France; 5 Department of Hematology, CHU de Limoges, Limoges, France; 6 Department of Hematology, Université Paris-Saclay, Hopital Bicêtre, Le Kremlin-Bicêtre, France; 7 Department of Clinical Hematology, Institut Curie, Saint Cloud, France; 8 Department of Infectious Diseases, Pitié-Salpêtrière Hospital, Assistance Publique-Hôpitaux de Paris (AP-HP) and Sorbonne Université, Paris, France; 9 Department of Neurology, AP-HP Nord, Université de Paris Cité, Saint-Louis Hospital, AP-HP, Paris, France; 10 Department of Neurooncology, CHRU de Nancy, Hôpital Central, Nancy, France; 11 Neuropathology Department, Pitié-Salpêtrière University Hospital, AP-HP, Paris, France; 12 Neurosurgery Department, Pitié-Salpêtrière University Hospital, AP-HP, Paris, France; 13 Neuro-Oncology Department, Pitié-Salpêtrière Hospital, Assistance Publique-Hôpitaux de Paris, Sorbonne Université, Inserm, CNRS, UMR S 1127, ICM, IHU, Paris, France; 14 INSERM, CNRS, Laboratory of Biomedical Imaging, Sorbonne University, Paris, France

**Keywords:** MRI, lymphoma, immunocompromised, HIV, solid organ transplantation, FDG-PET

## Abstract

**Background:**

Epstein–Barr virus (EBV)–associated primary central nervous system lymphoma (PCNSL) is a rare form of extranodal non-Hodgkin’s lymphoma closely linked to immunodeficiency. Imaging characteristics of EBV-associated are reported to differ from those of typical EBV-negative PCNSL. This study aims to describe the radiological and nuclear medicine imaging features in a large cohort of patients with EBV-associated PCNSL.

**Methods:**

We conducted a multicenter retrospective descriptive study between 2008 and 2025 on patients with a diagnosis of EBV-associated PCNSL. MRI variables and FDG-PET/CT uptake were assessed.

**Results:**

Fifty-eight cases of EBV-associated PCNSL were included. All but 1 patient were immunosuppressed. Multiple lesions were present in 71% of cases (41/58). Supratentorial involvement was observed in 90% of cases (52/58). Heterogeneous contrast enhancement was noted in 90% (52/58), with ring-like enhancement in 41% (24/58). Leptomeningeal enhancement occurred in 31% of cases (18/58), and within this group, 50% showed perivascular space enhancement. Lesions showed hypercellularity in 83% (48/58) and intralesional hemorrhage in 81% (47/58). An “eccentric target” sign was present in 26% of cases (15/58), while a “concentric target” sign in 14% (5/35). On FDG-PET, 25/30 patients had hypermetabolic lesions (25/30, 83%).

**Conclusion:**

Diagnosing EBV-associated PCNSL is challenging due to its rarity and the broad differential diagnosis. Multiple necrotic and hemorrhagic lesions are the most suggestive MRI feature of EBV-associated PCNSL. “Eccentric” and “concentric” target signs, typically associated with CNS toxoplasmosis, can be observed. FDG-PET often reveals hypermetabolic lesions that support a neoplastic diagnosis. Histological confirmation remains essential for confidently treating this tumor entity.

## Introduction

Primary central nervous system lymphoma (PCNSL) is a rare and aggressive extranodal non-Hodgkin’s lymphoma confined to the brain, eyes, spinal cord, and cerebrospinal fluid. In immunocompetent patients, the most common subtype-diffuse large B-cell lymphoma (DLBCL) typically presents with lesions in the periventricular regions, corpus callosum, and basal ganglia [1–8]. Radiologically, these lesions show intense, homogeneous contrast enhancement on post-contrast T1-weighted imaging, minimal hemorrhage or necrosis, and marked hypercellularity, reflected by T2-hypointensity, high diffusion restriction, and an elevated choline/N-acetyl aspartate ratio [9, 10]. Magnetic resonance spectroscopy (MRS) may also reveal elevated lipids and lactate, which is unusual in non-necrotic enhancing lesions. Perfusion imaging usually demonstrates no or only a mild increase in relative cerebral blood volume (rCBV), though higher values can occur, alongside increased blood–brain barrier permeability [11, 12].

In severely immunocompromised individuals, such as those with acquired immune deficiency syndrome (AIDS) or undergoing immunosuppressive therapy, PCNSL can arise in association with Epstein–Barr virus (EBV) infection [13–15]. This tumor entity is very rare, accounting for less than 10% of PCNSLs, which themselves represent about 1–5% of all primary CNS tumors [16]. EBV, which infects over 90% of the population, is typically asymptomatic in immunocompetent hosts but can lead to EBV-associated PCNSL in immunocompromised patients due to the loss of EBV-specific CD4 + T cells. Genetically, EBV-associated PCNSL lacks common mutations found in EBV-negative cases, such as MYD88, CD79B, or PIM1, and is now recognized as a distinct tumor entity in the WHO classification [15]. Its imaging features—including hemorrhage, central necrosis, and peripheral ring enhancement [17–21]––can resemble other conditions frequent in immunocomprised patients, particularly neurotoxoplasmosis [22–24], which also presents with ring-enhancing necrotic lesions and may show “eccentric” or “concentric” target signs [25, 26], described as pathognomonic.

Given the rarity of EBV-associated PCNSL, studies describing its radiological features are limited by small sample sizes, between 6 and 30 patients. Awareness of its imaging characteristics can facilitate timely diagnosis and treatment, potentially improving patient outcomes. This study aims to analyze the pre-therapeutic imaging findings of pathologically confirmed EBV-associated PCNSL.

## Methods

### Standard protocol approvals and patient consent

We conducted a multicenter retrospective study, including patients between September 2008 and February 2025 from five centers included in the LOC (lymphomes oculo-cérébraux), K-Virogref and/or CANCERVIH network databases. These are French national networks certified by the National Cancer Institute and dedicated to primary CNS lymphomas, viral-induced cancers following solid organ or hematopoietic stem cell transplantation and cancers in the setting of HIV infection, respectively. The databases were approved by the Institutional Ethical Committee of the coordinating center and by the French “Commission Nationale de l’Informatique et des Libertés” (CNIL). All the patients provided informed consent to participate in the database search and for the use of their data. This study was conducted in accordance with the Declaration of Helsinki.

### Enrollment of subjects

Inclusion criteria were as follows:

1. A diagnosis of EBV-induced primary central nervous system lymphoma;
2. Availability of a pre-therapeutic MRI, including at least contrast-enhanced T1-weighted and FLAIR sequences;
3. The absence of systemic lymphoma at baseline, confirmed by body computed tomography (CT) or 18F-FDG PET/CT.

EBV status was proven on brain tissue samples by in situ hybridization for Epstein-Barr virus-encoded small RNA, immunohistochemistry for EBV-related proteins or by polymerase chain reaction (PCR).

In cases where histopathological analysis confirmed B-cell lymphoma, but tissue samples were EBV-negative, subjects were included if they had both severe immunosuppression and evidence of EBV replication in cerebrospinal fluid (CSF).

One patient was included without brain tissue sample, based on a monotypic B-cell lymphoproliferation identified via lumbar puncture, positive EBV PCR in CSF, and a diagnosis of HIV infection with uncontrolled viral replication.

Demographic characteristics, immunological status, CSF IL-10 and IL-6 levels and clinical history of immunosuppression were recorded for each subject. CSF IL-10 and IL-6 levels were considered increased if superior to 4 pg/mL.

### Imaging

CT and MRI were retrospectively reviewed by a neuroradiologist with 5 years of neuro-oncology experience, one radiologist-in training and one neurologist. All imaging was performed before chemotherapy. One patient received high-dose corticosteroids prior to imaging; the other patients received either no steroids or mild doses (< 20 mg prednisone). Topographic variables based on the distribution of lesion burden were considered such as presence of singular or multiple lesions, unilateral or bilateral, and supratentorial or infratentorial distribution. In addition, for supratentorial lesions, we discriminated between cortical-juxtacortical and deep involvement. Within deep involvement, different distribution patterns were described: we specified whether the lesions were in the basal ganglia, periventricular regions, corpus callosum, or in deep white matter areas such as the corona radiata and centrum semiovale.

CT imaging of EBV-associated PCNSL lesions was classified as hyperdense, hypodense, or non-visible.

On MRI, a distinction between homogeneous and heterogeneous gadolinium contrast-enhancement of the lesions was made. In addition, within the heterogeneous pattern we differentiated between homogeneous enhancement, ring-like enhancement, a heterogeneous peripheral mass-like enhancement, and leptomeningeal enhancements. In the case of leptomeningeal enhancement, a further distinction was made as to whether the enhancement involved the perivascular spaces (Virchow–Robin spaces) [27]. In addition, the presence of “concentric target sign” and “eccentric target sign” was analyzed in accordance with previous studies [25, 28, 29]. The concentric target sign is defined by alternating concentric rings of hyperintensity and hypointensity on T2 sequences. The eccentric target sign consists of a ring-enhancing lesion with an eccentric nodular component attached to the inner margin of the ring, seen on contrast-enhanced T1 sequences. The presence or absence of intralesional hemorrhage was searched simultaneously on the available susceptivity imaging sequence (T2*, SWI, SWAN), on non-contrast T1-weighted images and on non-enhanced CT. Diffusion weighted imaging (DWI) and apparent diffusion coefficient (ADC) maps were also visually reviewed discerning between lesions with or without diffusion restriction in non-hemorrhagic tissue components. When available, advanced MRI studies such as perfusion weighted imaging (PWI), both arterial spin labeling (ASL) and dynamic susceptibility contrast (DSC) MR perfusion, as well as spectroscopy were also reviewed. Lesions were considered hyperperfused if leakage corrected rCBV was above 1.75 the contralateral normal appearing white matter.

All PET/CT images were reviewed by a nuclear medicine specialist. Lesions were classified as hypermetabolic or hypometabolic based on PET/CT findings. SUVmax and maximum tumor-to-background ratio (TBRmax) were calculated for each lesion. In cases of multiple lesions, the lesion with the highest metabolic activity was selected for analysis.

## Results

### Demographics, Table 1

**Table 1:** Clinical and biological characteristics

	*N* (%)
*N*	58
Age at diagnosis, median (min–max)	55 (16–77)
Sex: male/female	36 (62%)/22 (38%)
Cause of immunodepression	
HIV CD4 cells/mm^3^ in PLWH, median (min–max)	14 (24%) 37 (0–251)
Immunosuppressive therapy	42 (72%)
Solid organ transplantation Kidney transplantation	31 (53%) 20 (34%)
Auto-immune condition Inflammatory bowel disease SLE Myositis Autoimmune hepatitis	9 (16%) 4 (7%) 3 (5%) 1 (2%) 1 (2%)
Hematopoietic stem cell transplant	2 (3%)
CVID	1 (2%)
No immunodepression	1 (2%)
Immunosuppression duration before lymphoma diagnosis, median in years (min–max)	10 (0.5–34.7)
EBV markers on biopsy	55/57 (96%)
EBV replication on CSF Mean replication, in log	24/30 (80%) 3.2 (1.52 to 5)
EBV replication in blood sample Mean replication, in log	43/45 (96%) 3.4 (2.3 to 8)
CSF-to-plasma EBV DNA ratio > 1	9/19 (47%)
IL-10 increase in CSF* Mean value (min–max) IL-6 increase in CSF* Mean value (min–max) IL-6/IL-10 ratio > 1	9/24 (38%) 2.5 (0–27) 12/24 (50%) 3.5 (0–280) 10/14 (71%)

*CD4* Cluster of differentiation 4, *CSF* Cerebrospinal fluid, *CVID* Common variable immunodeficiency, *EBV* Epstein–Barr virus, *HIV* Human immunodeficiency virus, *max* Maximum, *min* Minimum, *PLWH* People living with HIV, *SLE* Systemic lupus erythematosus

*Positive value if superior to 4 pg/ml

A total of 58 cases of Epstein–Barr virus–associated PCNSL were collected between September 2008 and February 2025. The cohort included a majority of male patients (36/58, 62%) and had a median age of 55 years (range from 16 to 77). All but one patient was immunocompromised at the time of diagnosis.

Of the 57 patients with immunosuppressive conditions, 31 (53%) had a history of solid organ transplantation, 14 (24%) were HIV-positive, 9 (16%) were receiving immunosuppressive therapy for an autoimmune disorder, 2 (3%) had undergone hematopoietic stem cell transplantation, and 1 (2%) had a primary immunodeficiency (common variable immunodeficiency-CVID).

Among HIV-positive patients, the median CD4 count at diagnosis was 37 cells/mm^3^. For patients with solid organ transplants or autoimmune conditions, the median duration of immunosuppressive therapy prior to diagnosis was 10 years.

Fifty-seven patients underwent brain biopsy. A positive EBV status was found on 55/57 (96%) brain tissue samples. Two patients had histopathological confirmation of B-cell lymphoma with negative EBV status on biopsy; both had EBV replication found in the CSF. The patient who did not undergo brain biopsy was included based on the identification of a monotypic B-cell lymphoproliferation in CSF obtained by lumbar puncture, along with a positive EBV PCR result in the CSF (Table 2).

**Table 2:** Imaging features, *non-mutually exclusive

	*N* (%)
Number of patients	58
Multiple lesions	41 (71%)
Lesion distribution	
Infratentorial only	6 (10%)
Supratentorial only	37 (64%)
Both	15 (26%)
Distribution of supratentorial lesions, (*N* = 52)	
Superficial only	15 (26%)
Deep only	16 (28%)
Both	21 (36%)
Distribution of deep lesion sites*	
Basal ganglia	23 (40%)
Periventricular regions	18 (31%)
Corpus callosum	13 (22%)
Corona radiata and centrum semiovale	7 (12%)
Contrast-enhanced T1 pattern	
Ring	
Mass like Smooth ring	28 (48%) 24 (41%)
Homogeneous	6 (10%)
Leptomeningeal enhancement Virchow–Robin enhancement	18 (31%) 9 (16%)
Eccentric target sign Concentric target sign	15 (26%) 5/35 (14%)
Presence of hemorrhage	47 (81%)
Diffusion restriction	48 (83%)
Perfusion weighted imaging	
Hypoperfusion Hyperperfusion Blood–brain barrier rupture	21/24 (88%) 3/24 (13%) 14/24 (58%)
Presence of a lipid peak on spectroscopy	11/13 (85%)
Hyperdense lesions on CT	31/37 (84%)
Lesion uptake on PET SUVmax (min–max) TBRmax (min–max)	25/30 (83%) 11 (5.4–63.1) 1.7 (1.1–5.3)

*CT* Computed tomography, *PET* Positron emission tomography, *PWI* Perfusion weighted imaging, *SUVmax* maximum standardized uptake value, *TBRmax* maximum target-to-background ratio

### Imaging features, table 2 

#### Topography

**Fig. 1:**
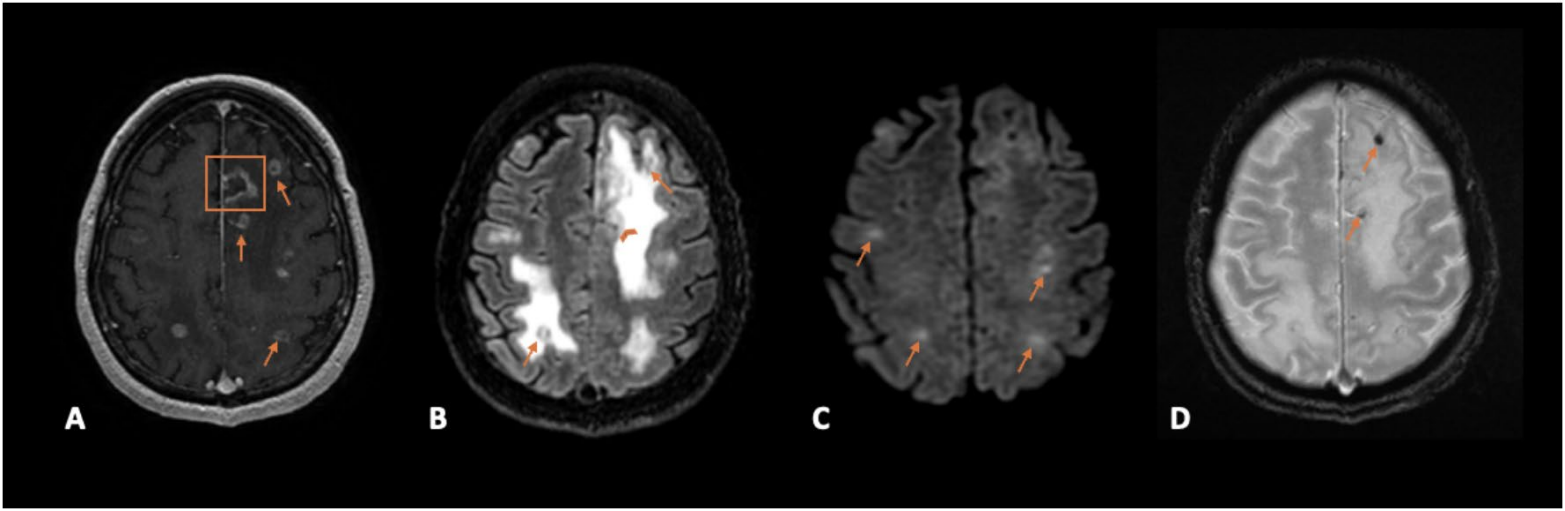
Example of a patient with EBV-associated brain lymphoma. **A** Multiple lesions demonstrating ring enhancement (arrow) and necrotic, heterogeneous enhancement (square), all with a cortico-subcortical distribution; **B** On FLAIR imaging, the lesions appear hypointense (arrow) or isointense (arrowhead), accompanied by significant perilesional edema; **C** Diffusion-weighted imaging (DWI) with a b-value of 1000 (b1000) shows restricted diffusion within the lesions (arrow); **D** T2-weighted gradient echo imaging reveals hemorrhagic components in some of the lesions (arrow)

Out of 58 cases, 41/58 (71%) had multiple lesions. Supratentorial involvement was found in 52/58 (90%) of cases, with an association of supratentorial and infratentorial lesions in 15/58 (26%) of patients. A representative subject with typical EBV-associated brain lymphoma is presented in Fig. 1.

**Fig. 2:**
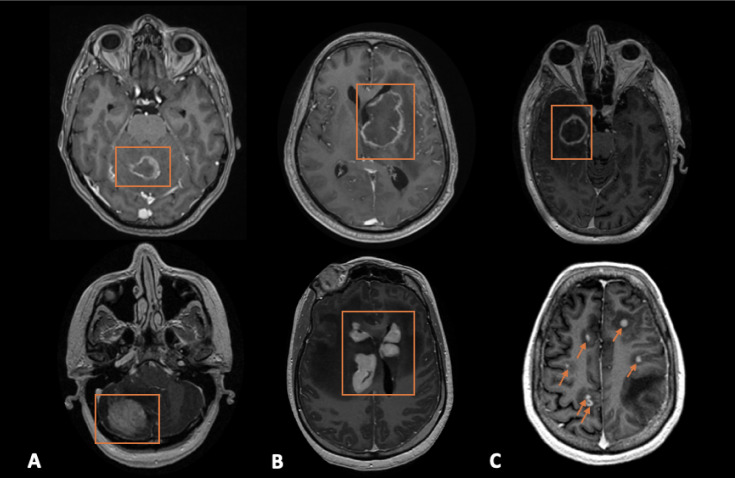
Examples of lesion distribution on contrast-enhanced T1-weighted sequences. **A** Infratentorial location, upper row: necrotic lesions, lower row: homogeneous lesions; **B** Deep location (basal ganglia and periventricular region), upper row: necrotic lesions, lower row: predominantly homogeneous lesions; **C** Peripheral location, upper row: necrotic lesions, lower row: predominantly homogeneous lesions

Isolated cortical-subcortical and central distribution were found in 15/58 (26%) and 16/58 (28%) of patients, respectively. basal ganglia and periventricular regions were the most frequently central locations involved (23/58, 40% and 18/58, 31% respectively), followed by corpus callosum (13/58, 22%). White matter regions such as corona radiata and centrum semiovale were affected in seven cases (12%). The topography distribution of the lesions is illustrated in Fig. 2.

#### Contrast enhancement

**Fig. 3:**
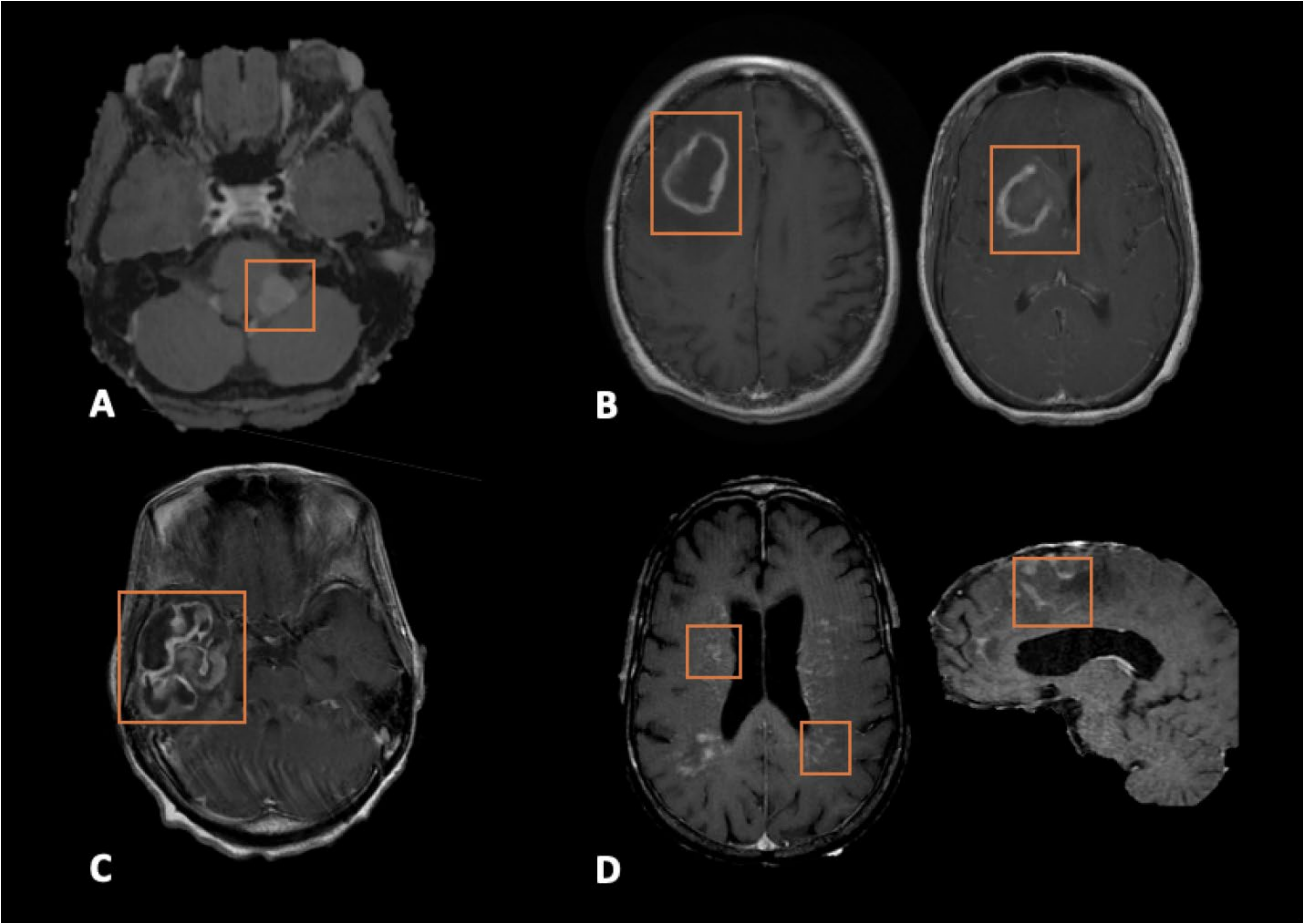
Examples of enhancement types on contrast-enhanced T1-weighted sequences. **A** Homogeneous enhancement. **B** Ring enhancement (left: complete ring, right: open ring); **C** Necrotic, heterogeneous enhancement. **D** Leptomeningeal enhancement (left: Virchow–Robin enhancement, right: leptomeningeal)

All cases demonstrated contrast enhancement on MRI. The majority (52/58, 90%) exhibited heterogeneous enhancement. Among these, 24/58 patients (41%) had at least one lesion with a smooth, ring-like enhancement, and 28/58 (48%) had at least one necrotic lesion with irregular peripheral enhancement. Leptomeningeal enhancement was observed in 18/58 cases (31%), including 9 cases where the enhancement involved the Virchow–Robin perivascular spaces. Figure 3 synthesizes contrast enhancement patterns.

**Fig. 4:**
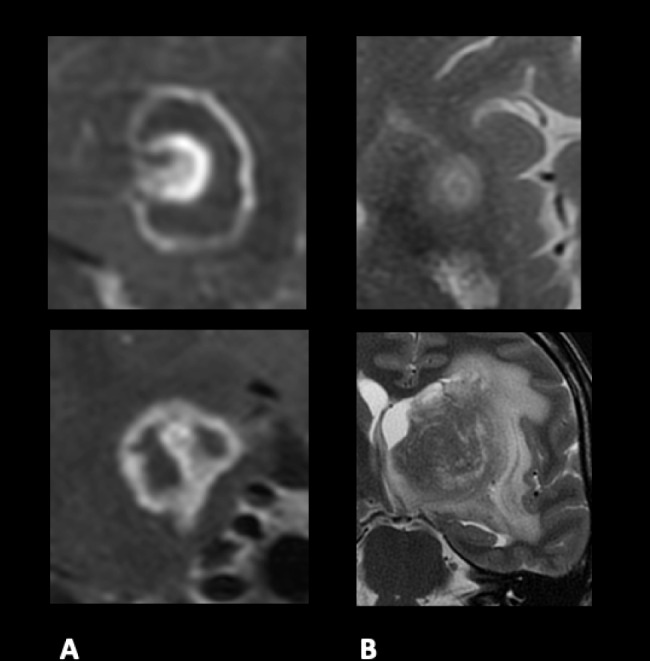
Examples of “eccentric” and “concentric” target signs in EBV-associated brain lymphoma. **A** “Eccentric target” signs on contrast-enhanced T1-weighted sequences; **B** “Concentric target” sign on T2-weighted sequences

An eccentric target sign was seen in 26% of cerebral EBV-associated PCNSL cases (15/58). The “concentric target” sign was strictly evaluated in T2-weighted images and was visible in 14% of the cases in which this sequence was available (5/35). Four cases had both signs at the same time. Figure 4 illustrates two representative cases of the “eccentric” and “concentric” target signs in our population.

#### Advanced MRI, CT, and FDG-PET imaging features

Intralesional hemorrhage was commonly observed, occurring in 47 out of 58 (81%) of EBV-associated PCNSL cases. DWI showed diffusion restriction outside of a hemorrhagic region in 48/58 (83%) of cases. Diffusion restriction and hemorrhage usually followed or were closely associated with the contrast-enhanced lesion.

Twenty-five patients underwent PWI. DSC relative cerebral blood volume corrected (rCBVc) maps showed hypoperfusion in 21/24 cases (88%) and only 3 cases showed hyperperfusion (13%). DSC K2 leakage maps showed blood–brain barrier disruption in 58% of cases (14/24).

Thirteen cases had a spectroscopy with both short (35 ms) and long (135 ms) time of echo (TE). All had an increased Cho/Naa ratio and all but two had elevated lipids with a characteristic resonance peak at about 1.3 ppm observed in both short and long TE. One case had a lactate chemical shift peak inversion in long TE.

CT was available for 37 subjects. Thirty-one out of 37 cases (84%) presented hyperdense intraparenchymal lesions.

**Fig. 5:**
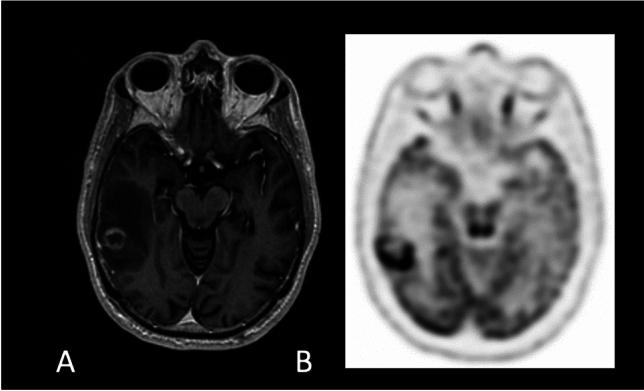
Example of hypermetabolism in EBV-associated brain lymphoma. **A** Ring-enhancement lesion on contrast-enhanced T1-weighted sequences; **B** Hypermetabolism on PET-CT

Metabolic imaging with PET/CT was available for 30 patients. The majority (25/30, 83%) demonstrated hypermetabolic lesions. The median SUVmax of the most avid lesion was 11 (range, 5.4–63.1). To account for variability between imaging systems, TBRmax was calculated; the median TBRmax was 1.7 (range, 1.1–5.3). An example is shown in Fig. 5.

## Discussion

This study presents the largest cohort to date analyzing the radiological characteristics of EBV-associated PCNSL. Our findings confirm that patients with this pathology frequently exhibit multiple ring-enhancing necrotic-hemorrhagic lesions in both superficial and deep locations, as previously described. Additionally, our results reaffirm that immunosuppression is almost universally present in this tumor entity. Building on previous research, our study highlights a high prevalence of leptomeningeal and perivascular enhancement—greater than that reported in non-immunocompromised PCNSL [3, 30]. For the first time, we also assessed the specificity of the eccentric and concentric target signs in a non-infectious context.

In immunocompromised patients, the presence of cerebral ring-enhancing lesions often raise suspicion of an infectious disease, particularly when associated with leptomeningeal enhancement. Neurotoxoplasmosis is the most common differential diagnosis and the most frequently reported neurological opportunistic infection in patients with HIV [31]**.** Neurotoxoplasmosis also presents as superficial and/or deep ring-enhancing single or multiple lesions and is frequently suspected when eccentric or concentric target signs are present on imaging. In particular, the concentric target sign was previously considered pathognomonic for cerebral toxoplasmosis [25]. In our population, the concentric target sign was observed in 14% of cases, and the eccentric target sign in 26% of cases, confirming the overlap in MRI features between EBV-associated PCNSL and neurotoxoplasmosis.

Hemorrhagic remnants are frequent in both entities, reported in 93% of EBV-associated PCNSL patients [24] and in 81% of our population. While hypercellularity beyond hemorrhagic regions was commonly observed in our cohort (83%)—consistent with earlier reports of more extensive diffusion in toxoplasmosis lesions compared to lymphoma [32]— ADC ratios remain insufficient on their own to reliably differentiate between the two conditions [33].

Neo-angiogenesis was rarely observed in our population (16%), while blood–brain barrier disruption was prominent, mirroring observations in diffuse large B-cell lymphoma in immunocompetent individuals [10, 11]. However, these features alone do not provide a dependable means of distinguishing lymphoma from neurotoxoplasmosis [22]. Lipids were also often detected but are non-specific in necrotic lesions, and are also found in toxoplasmosis [34]. Consequently, conventional and advanced MRI sequences alone do not allow for a definitive radiological diagnosis.

PET/CT may be a useful tool for differentiating neurotoxoplasmosis from PCNSL, as neurotoxoplasmosis typically does not demonstrate marked hypermetabolism [23], while hypermetabolic uptake on FDG PET/CT was observed in 83% of patients, consistent with previously published studies [23], with SUV values between 5.4 and 63.1. Several studies have evaluated the utility of FDG PET/CT for distinguishing toxoplasmosis from PCNSL in patients with acquired immunodeficiency syndrome, reporting SUV values below 10 in neurotoxoplasmosis and above 10 in PCNSL. However, these studies included small patient cohorts, limiting the ability to define a reliable SUV cutoff.

Blood and CSF EBV PCR can also be useful to suspect PCNSL. In the largest study of primary CNS post-transplant lymphoproliferative disease [14], EBV DNA was detected in only 30% of patients in whole blood or CSF, despite EBV being present in 94% of brain biopsy specimens. By contrast, in HIV-associated PCNSL, detection of EBV DNA in CSF appears substantially higher, with reported sensitivities ranging from 63 to 97%, depending on the threshold used [35]. In our study, EBV replication was detected in 96% of blood samples and in 80% of CSF samples. Evidence of *Toxoplasma* replication should be sought, as it demonstrates excellent specificity but only moderate sensitivity (50–80%) [31].

Interleukin-10 has been described as a valuable diagnostic marker and potential prognostic biomarker in PCNSL among immunocompetent patients [36]. In our cohort, however, IL-10 elevation was observed in only 42% of patients with available measurements, substantially lower than the 80–90% rates reported in immunocompetent populations. In contrast, IL-6 levels were more frequently elevated, with 71% of patients demonstrating higher IL-6 concentrations. This discrepancy may reflect differences in tumor biology in our population, including EBV–related upregulation of IL-6 through NF-κB activation [37], or a stronger contribution of necrosis-associated inflammatory responses. Additionally, the lower prevalence of IL-10 elevation may be related to a smaller overall tumor burden compared to immunocompetent patients.

Interestingly, cases of coexisting PCNSL and neurotoxoplasmosis have been described in the literature [38] and may contribute to overlapping clinical or imaging features reported in some studies; however, no such cases were included in the present study.

Due to the non-specific imaging features of EBV-associated lymphomas, histopathological diagnosis via brain biopsy is essential when single or multiple necrotic ring-enhancing lesions are present, especially in urgent settings and in cases of *Toxoplasma gondii* seronegativity. Biopsy should also be considered if there is a poor response to empirical treatment for neurotoxoplasmosis, to avoid missing a potential coexistence of lymphoma [39]. Empirical treatment for neurotoxoplasmosis should only be initiated in cases of positive *Toxoplasma gondii* serology and/or positive *Toxoplasma gondii* PCR in the CSF, and concurrently, after exclusion of lymphoma via a negative CSF broad panel that includes cytology, immunophenotyping, clonality, and interleukin analysis. In this context, it is important to note that patients may develop neurotoxoplasmosis after allogeneic transplantation even without positive serology.

**Fig. 6:**
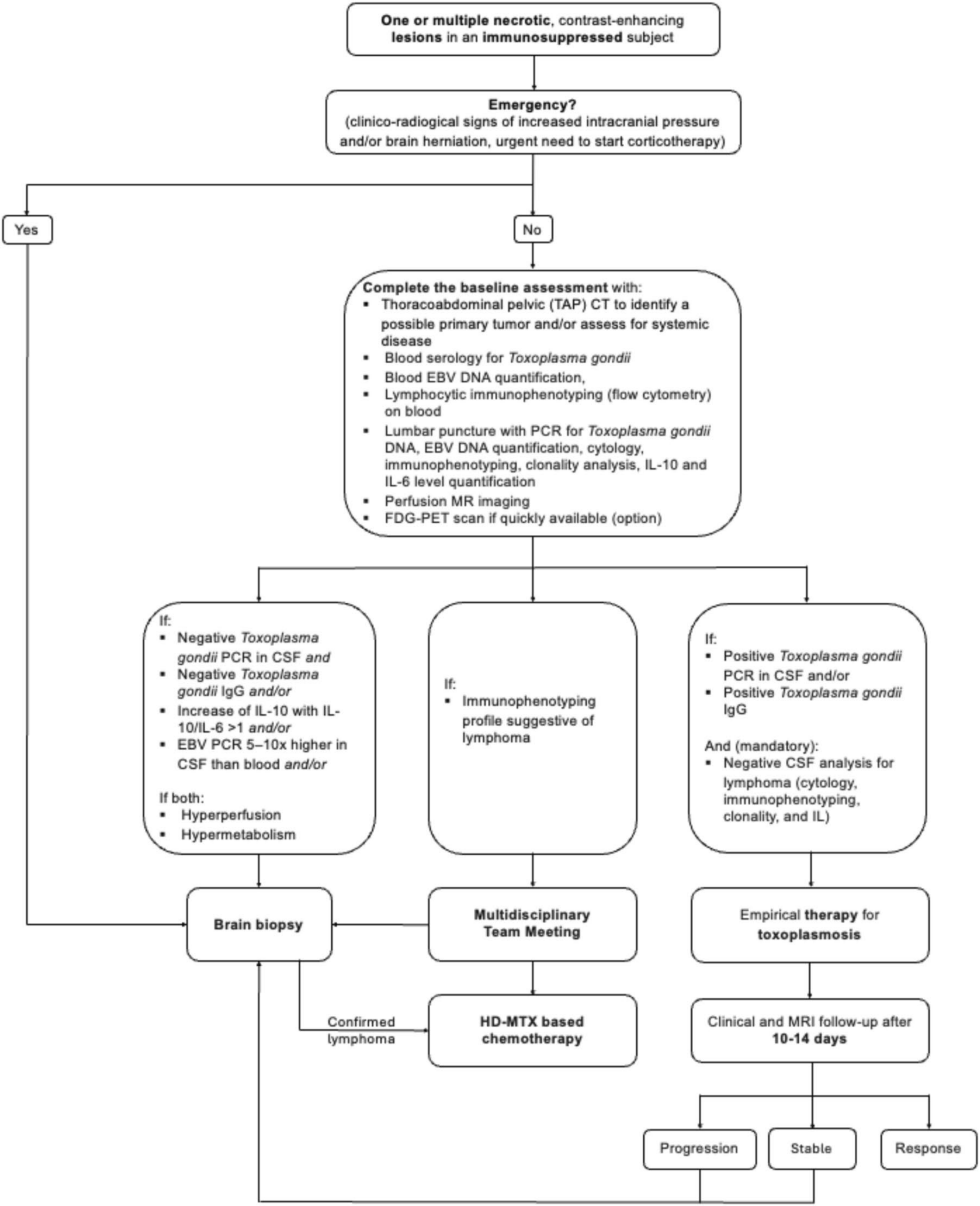
Suggested diagnostic algorithm. *CSF* Cerebrospinal fluid, *CT* Computed tomography, *DNA* Deoxyribonucleic acid, *EBV* Epstein–Barr virus, *IL* Interleukin, *PCR* Polymerase chain reaction

To conclude, distinguishing between EBV-associated lymphomas and neurotoxoplasmosis is difficult, as these two entities share multiple imaging features. To address this clinical complexity, a diagnostic algorithm for managing contrast-enhancing lesions in immunosuppressed patients is proposed in Fig. 6. This flowchart is inspired by the first proposal from Lancet [31] (2012) and has been updated based on clinical evidence and practical expertise. Its main goal is to increase awareness of EBV-associated lymphomas, as sustained clinical responses are possible even when therapeutic responses present with atypical imaging findings, as recently demonstrated [40].

Our study has the inherent limitations of a retrospective design. Not all MRI sequences were available for every patient, which may have led to an incomplete evaluation of the lesions. MRI images had heterogeneous parameters and after contrast injection, some patients had gradient-echo, whereas others had spin-echo T1 sequences. Moreover, the magnetic field was also not homogeneous (1.5 and 3 Tesla).

## Conclusion

The radiological characteristics of EBV-associated PCNSL differ clearly from those of classical immunocompetent EBV-negative lymphomas but remain non-specific. Both eccentric and concentric target signs may be present. FDG PET/CT may represent a useful noninvasive tool for differentiating central nervous system lymphoma from neurotoxoplasmosis. Our findings support the discussion of brain biopsy, especially in cases of *Toxoplasma gondii* seronegativity.

## Data Availability

The datasets used and/or analyzed during the current study are available from the corresponding author upon reasonable request.
